# Supplementary material for: Comparison of Saliva and Midturbinate Swabs for Detection of SARS-CoV-2
Source: Microbiol Spectr. 2022 Mar 21;10(2):e00128-22. doi: 10.1128/spectrum.00128-22 (PMC9045394; doi:10.1128/spectrum.00128-22)
Supplement: SUPPLEMENTAL FILE 1 — Supplemental material. Download SPECTRUM00128-22_Supp_1_seq6.pdf, PDF file, 0.4 MB [file spectrum00128-22_supp_1_seq6.pdf]

Supplementary Information for

Comparison of Saliva and Mid-Turbinate Swabs for Detection of SARS-CoV-2

Jianyu Lai, B.Med., M.P.H.[a,b], Jennifer German, Ph.D.[b], Filbert Hong, Ph.D.[b], S.-H. Sheldon Tai, Ph.D.[b], Kathleen M. McPhaul, Ph.D., M.P.H.[b], Donald K. Milton, M.D., Dr.P.H.[b] for the University of Maryland StopCOVID Research Group[*]

[a] Department of Epidemiology and Biostatistics, University of Maryland School of Public Health, College Park, Maryland, USA

[b] Public Health Aerobiology and Biomarker Laboratory, Institute for Applied Environmental Health, University of Maryland School of Public Health, College Park, Maryland, USA

Supplemental Tables

Table S1. Association between detection frequency and sample types (MTS vs. saliva) [a]

Sample Positive	*Sample Type*		***Total***
	MTS	Saliva	
No	171	168	339
Yes	29	32	61
Total	200	200	400

[a] χ^2=0.077, df=1, p=0.781

Table S2. MTS and saliva for positive participants (N=14)

	Number of subjects	Number of samples	Number of positive samples, N (%)	Ct value of positive samples, median (range)[a]	GM (95% CI)[b] of all samples	GM (95% CI)[c] of positive samples
MTS	13	41	29 (71)	20 (12, 33)	2.2×10^5 (1.0×10^5, 4.9×10^5)	4.5×10^6 (3.5×10^6, 5.9×10^6)
Saliva	12	41	32 (78)	27 (16, 35)	2.3×10^3 (1.2×10^3, 4.3×10^3)	4.5×10^5 (4.0×10^5, 5.0×10^5)

a. A positive sample was defined as having Ct < 40 in PCR detection for at least two out of three SARS-CoV-2 genes (N, S, ORF1ab)

b. GM = geometric mean. The GMs were computed, accounting for samples below the LOD, using a linear mixed-effects model for censored responses (R Project LMEC package) using data for all samples of each sample type with nested random effects of samples within study participant.

c. GM were calculated using data from only positive samples of each sample type with nested random effects of samples within study participant.

Table S3a. Viral RNA detection in paired saliva and MTS samples from participants with one or more positive samples (N=14) [a]

Saliva Positive	*MTS Positive*		***Total***
	No	Yes	
No	6	3	9
Yes	6	26	32
Total	12	29	41

[a] Cohen's Kappa between the two sample types was calculated as κ=0.43

Table S3b. Viral RNA detection in paired saliva and MTS samples from symptomatic participants with one or more positive samples (N=13) [a]

Saliva Positive	*MTS Positive*		***Total***
	No	Yes	
No	6	3	9
Yes	6	25	31
Total	12	28	40

[a] Cohen's Kappa between the two sample types was calculated as κ=0.42

Table S4. Saliva and MTS testing results for the asymptomatic positive participant (N=1)

	**Ngene Ct**	**Orf1ab Ct**	**Sgene Ct**	**Average Ct**
**MTS**	26.7	25.2	25.6	25.8
**Saliva**	33.4	36.3	34.5	34.7

Supplemental Figures

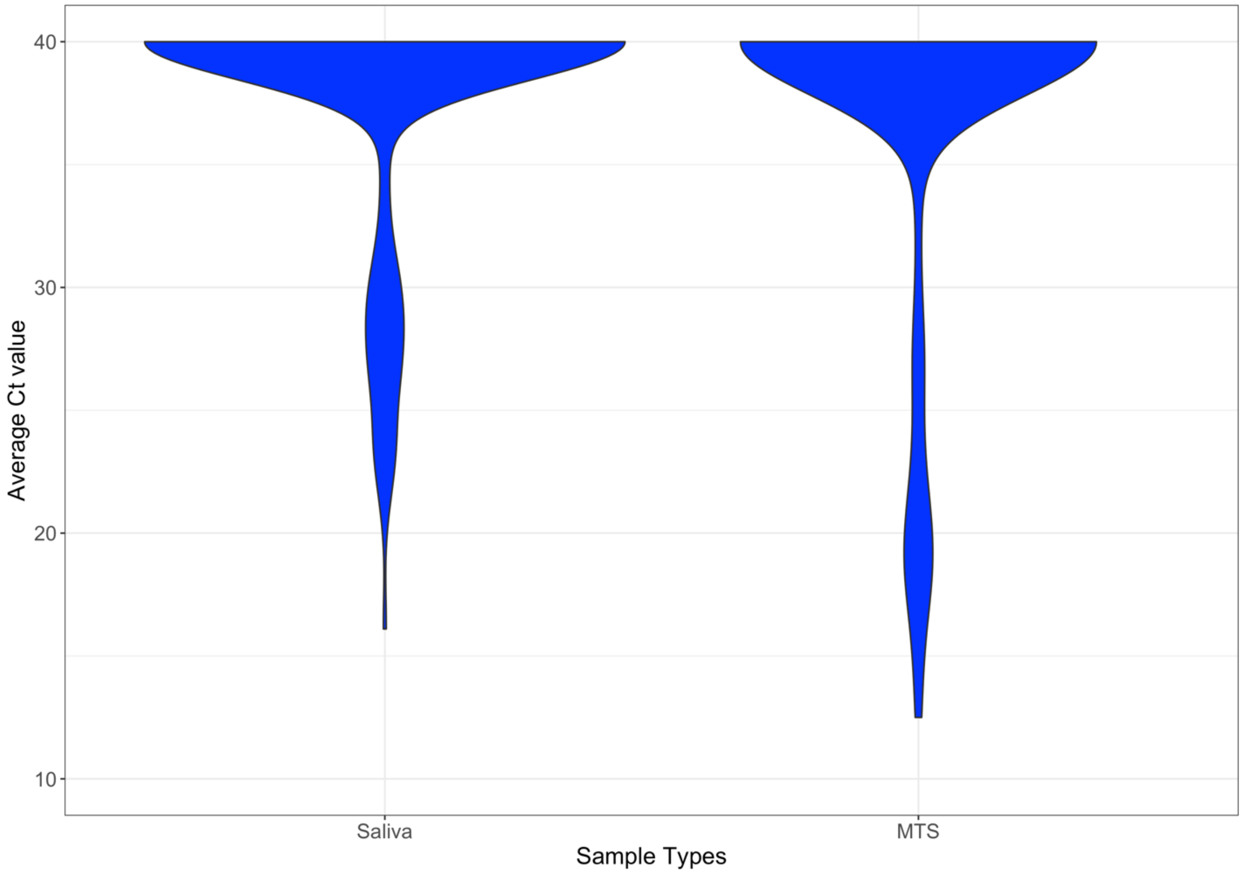

Figure S1. Distribution of Ct values by sample types among 58 participants and 400 samples.

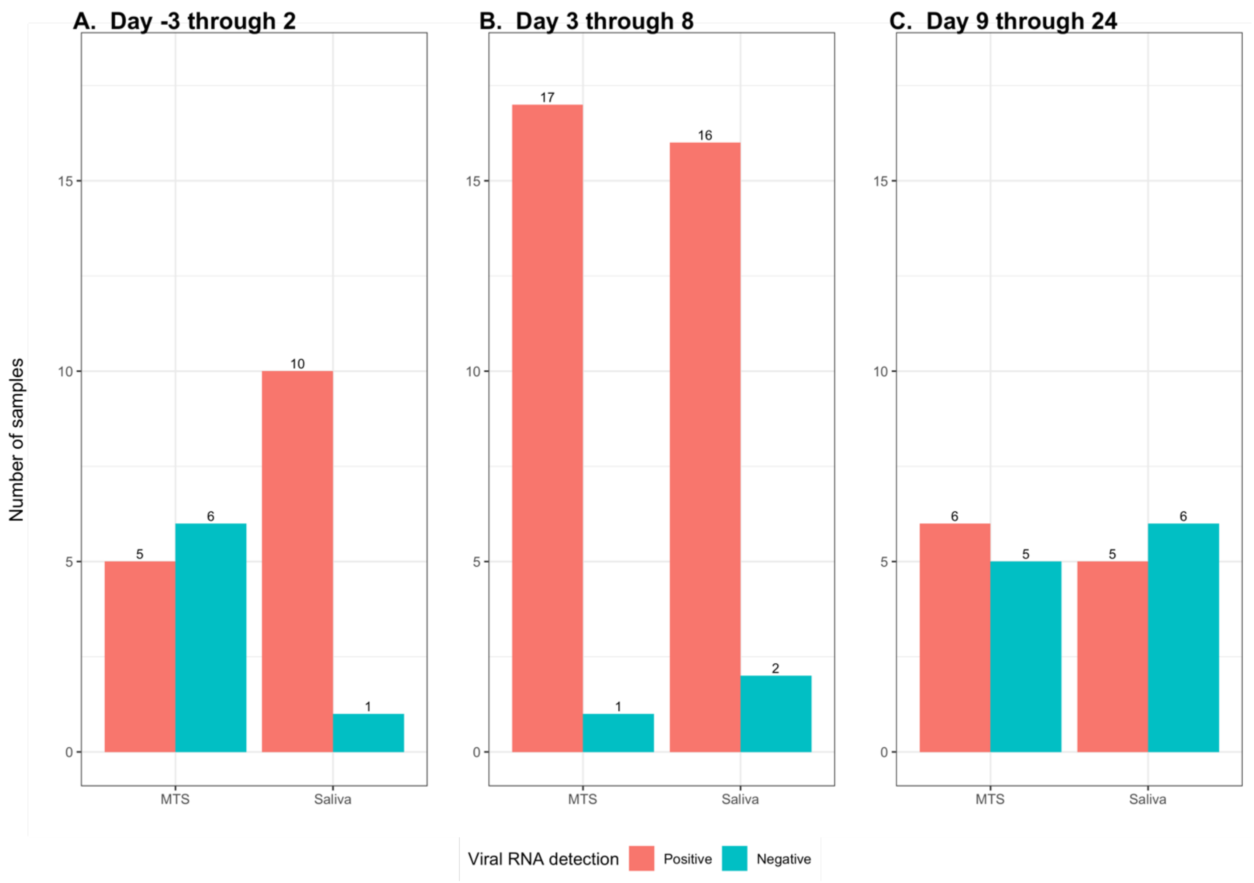

Figure S2. Distribution of samples from 13 symptomatic positive cases by days since symptom onset. A) Days -3 through 2, B) Days 3 through 8, C) Days 8 through 24.
